# Reconciling biome-wide conservation of an apex carnivore with land-use economics in the increasingly threatened Pantanal wetlands

**DOI:** 10.1038/s41598-021-02142-0

**Published:** 2021-11-23

**Authors:** Fernando R. Tortato, Rafael Hoogesteijn, Allison L. Devlin, Howard B. Quigley, Fábio Bolzan, Thiago J. Izzo, Katia M. P. M. B. Ferraz, Carlos A. Peres

**Affiliations:** 1 Panthera, 8 West 40th Street, 18th Floor, New York, USA; 2 Departamento de Ecologia, Instituto de Biologia, Universidade Federal Do Mato Grosso Do Sul, Campo Grande, MS Brazil; 3 Departamento de Ecologia e Botânica, Universidade Federal de Mato Grosso, Cuiabá, Mato Grosso Brazil; 4 Forest Science Department, Luiz de Queiroz College of Agriculture, University of São Paulo, Piracicaba, SP Brazil; 5 Centre for Ecology, Evolution and Conservation, School of Environmental Sciences, University of East Anglia, Norfolk, Norwich, UK; 6 Wildlife Biology Program, W.A. Franke College of Forestry and Conservation, University of Montana, Missoula, MT 59812 USA; 7 Instituto Juruá, Rua das Papoulas 97, Manaus, Brazil

**Keywords:** Conservation biology, Environmental economics, Fire ecology, Wetlands ecology

## Abstract

Conservation of carnivores involves finding solutions to minimize habitat loss and human-wildlife conflict. Understanding the nature of land-use economics can allow us to mitigate both threats. In the Pantanal, the two main economic activities are cattle ranching and ecotourism, each of which directly and indirectly affect the persistence of jaguars (*Panthera onca*). To understand how the geography of these economic activities is related to jaguar populations, we developed a jaguar distribution model (JDM), livestock density model, and ecotourism lodge density model for the Pantanal. Due to the recent wildfires within the Pantanal, we also assess the impact of burnt areas that are suitable for jaguars, cattle ranching, and tourism. Our JDM indicate that 64% of the Pantanal holds suitable habitat for jaguars. However, jaguar habitat suitability was positively correlated with ecotourism, but negatively correlated with areas most suitable for intensive cattle-ranching. This demonstrates a biome-wide scenario compatible with jaguar conservation. Of particular concern, recent wildfires overlap most suitable areas for jaguars. If wildfires become increasingly frequent, this would represent a serious threat to jaguars and many other wildlife populations. We emphasize the global importance of the Pantanal wetland ecoregion as a key stronghold for long-term jaguar conservation.

## Introduction

The often synergistic effects of human-carnivore conflict and land-use transformation are leading threats for the world’s remaining large felid populations^1^. Persistence of these species requires an understanding of their spatial relationships with the biophysical, social, and economic interfaces of potential conservation landscapes^2–4^. In many cases, solving human-wildlife conflict involves excluding large felid populations from fenced landholdings and establishing strictly protected areas^5^. However, including unprotected areas exposed to different land-uses will become increasingly essential for the future conservation of wildlands^6^.

Land use transformation represents the main threat to the conservation of the jaguar (*Panthera onca*), the largest cat in the Americas. The jaguar occupies 51% of its original range^7^, and intact habitat is increasingly lost to deforestation (i.e. conversion into cropland and pasture) and wildfires^4,8^. Jaguars prefer forested landscapes and display different resource selection patterns according to the environment, and exhibit some plasticity to adapt to man-made environments, but this increases the risk of human-jaguar conflict^9^.

The Pantanal of central South America is the world’s largest continental wetland, and is widely considered a key conservation ecoregion for jaguars^10,11^. For over two centuries, land use in the Pantanal has been focused on extensive ranching of rustic cattle breeds at low stocking densities on native pastures, which are managed according to the annual hydrological cycle of floodwaters and droughts^12,13^. Over the past three decades, a growing ecotourism industry has increasingly shared the landscape with traditional cattle ranching. Ecotourism has expanded throughout the Pantanal, and more recently, lucrative tourism focused on jaguar observation has increased in different regions of the Pantanal^14,15^.

Historically, retaliatory killings in cattle ranches has been the main threat to jaguars in the Pantanal^16^. However, the recent increase in ecotourism has added value to the species^17^ and has become an option to reconcile economic activities with jaguar conservation. Historically sustainable low-yield livestock ranching is increasingly challenged by competing forms of land-use, including the introduction of exotic pastures following deforestation of upland areas and subsequent intensification of bovine stocking rates^12,13,18,19^. More recently, wildfires have posed an additional serious threat to wildlife populations in the Pantanal. In 2020, the Pantanal experienced the worst drought in 50 years, and consequently the landscape became more vulnerable to wildfires, affecting biodiversity and economic activities such as cattle ranching^20^. The wildfires of 2020 resulted in the mortality of millions of vertebrates and directly impacted the biodiversity of the Pantanal, including its megafauna such as the jaguar^21^. This was a year of record-breaking wildfires, which burnt an area of over 4 million hectares, equivalent to one-third of the entire Pantanal biome. Such extreme events are alarming given that 43% of all burnt areas had no previous fire records over the past 20 years^22^. Regions affected by these fires included important protected areas for jaguar conservation, including the Encontro das Águas State Park^23^.

The increasing number and complexity of threats have led to a debate about the importance of maintaining the rather benign historically traditional Pantanal land-use system of extensive cattle ranching, deploying preventive measures against wildfires^21,22^, and how best to reconcile realistic economic alternatives with ecosystem conservation^13,18,24^. Understanding the relationship between jaguars, cattle ranching, and ecotourism in private landholdings is critical, especially since only 7% of the 140,000 km^2^ Pantanal biome in Brazil is designated as protected areas^13^. The remaining 93% is comprised of private landholdings, 80% of which are largely allocated to low-yield bovine cattle husbandry^12^. Identifying suitable areas for viable jaguar populations is therefore necessary to inform land stewardship, and landscape-scale conservation planning and decision-making. Species distribution models (SDMs) represent a useful tool to address these questions and help in fine-tuning target species conservation plans at vast landscape scales^25^, such as the entire Pantanal. Beyond the three categories (cattle ranching; ecotourism; and suitable habitat areas for jaguars), it is urgent to understand how the likely proliferation of wildfires may compromise long-term jaguar conservation plans.

Here, we quantitatively assess the economic landscape and occurrence of wildfires across the entire Pantanal biome and their relationships with jaguar conservation. More specifically, we build a jaguar distribution model (JDM) for the entire Pantanal biome to identify suitable habitat areas for jaguars, and develop two additional models to describe the (1) density of ecotourism lodges and (2) density distribution of bovine cattle. Finally, we intersect the three layers with areas affected by the most recent wildfires mapped over the last four years (2017–2020). With the two layers of economic use and probability of jaguar presence, we assess the spatial correlation of areas most suitable for jaguars with the intensity of economic use. We discuss the implications of our findings to develop conservation measures that can integrate the imperatives of land-use revenues and preventive measures against wildfires with the conservation of an iconic flagship species for the Pantanal, the jaguar.

## Results

### Jaguar distribution model

**Figure 1:**
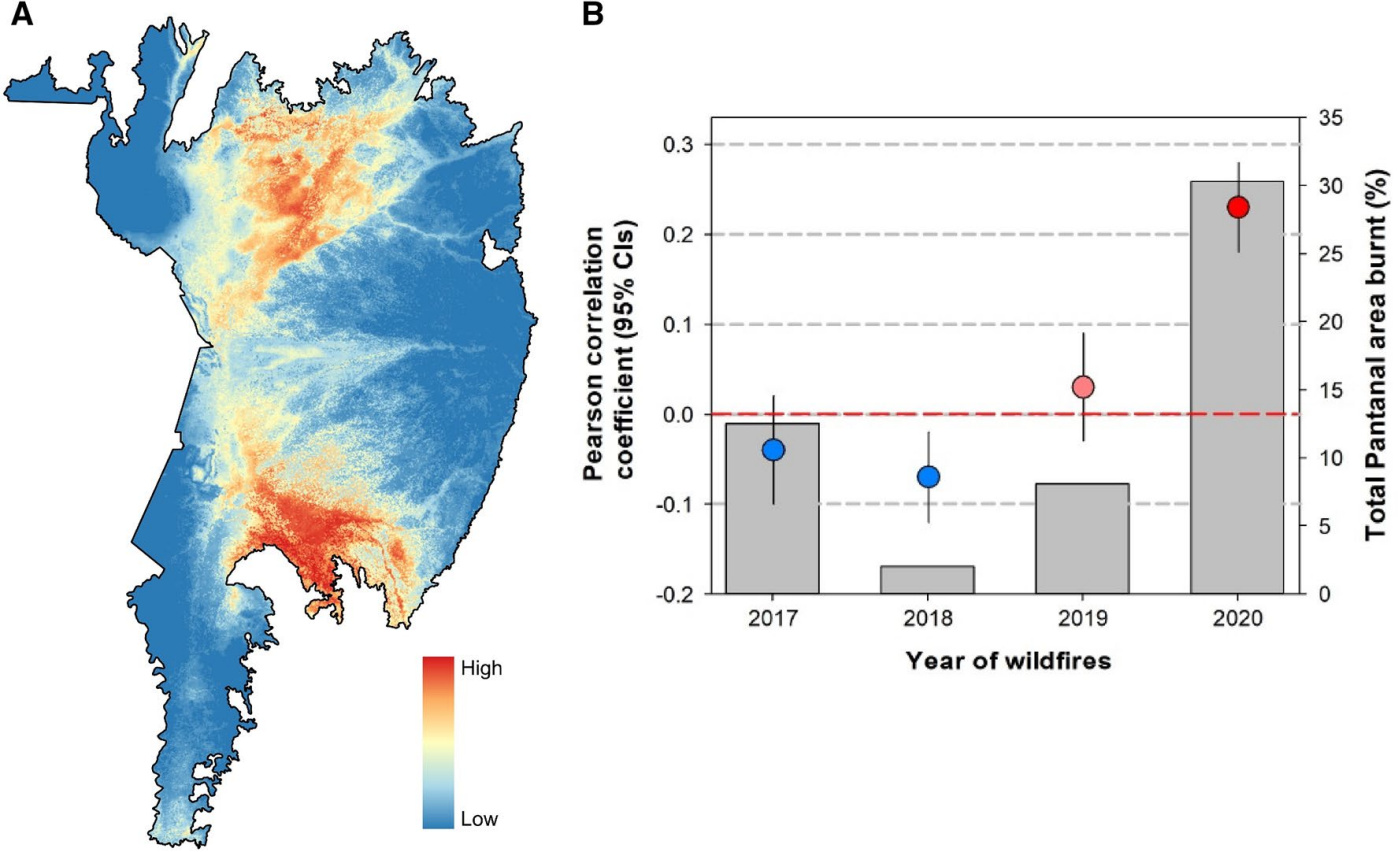
(**A**) Jaguar distribution model (JDM) across the Pantanal wetlands biome within Brazil, with red and blue areas colour-coded as high to low probability of jaguar occurrence, respectively. Map made in QGIS (v. 3.16.5; QGIS Development Team 2021). (**B**) Pearson correlation coefficient between habitat suitability for jaguars and the areas affected by wildfires in the last four years (2017–2020).

Our jaguar distribution model performed relatively well (AUC = 0.82 ± 0.03) revealing that, at present, some 64% (~ 90,000 km^2^) of the entire Brazilian Pantanal region is comprised of suitable to highly suitable habitat for jaguars (Fig. 1A). The environmental covariates that best explained jaguar occurrence across the entire biome included maximum temperature of the warmest month (*bio5*; 28.49%), elevation (22.89%), and land cover (19.44%). The model also identified two habitat regions that were particularly suitable (suitability > 0.40) for jaguar occurrence, which are located in the northern and southern portions of the Pantanal (Fig. 1A).

### Jaguar distribution and their relation with the economic landscape

**Figure 2:**
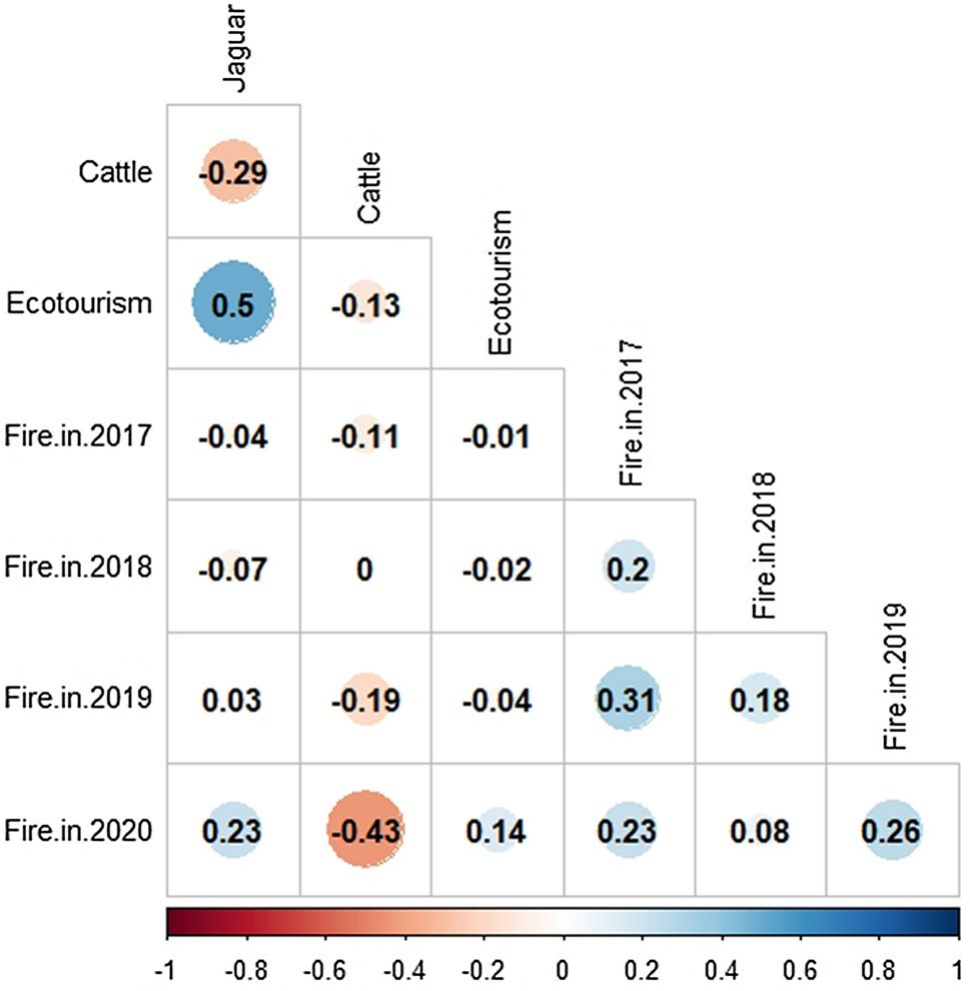
Correlation matrix between jaguar habitat suitability (Jaguar), cattle stocking density (Cattle), lodge density (Ecotourism), and incidence of annual burnt areas (Fire) between 2017 and 2020 in the Brazilian Pantanal.

As expected, there was a significant overall positive spatial correlation between jaguar habitat suitability and ecotourism potential as expressed by the geographic distribution of financially viable lodges (r = 0.50, *p* < 0.001, Fig. 2). In contrast, there was a significant overall negative spatial correlation between jaguar habitat suitability and areas most suitable for bovine livestock as expressed by cattle stocking density (r =  − 0.29, *p* < 0.001, Fig. 2) (see Supplementary material). These correlative trends were further supported when we compared observed differences in suitability values at the scale of hex-cells with those extracted from the null model (see Methods), with observed values falling well outside the respective null distributions (*p* < 0.0001). This indicates minimum spatial congruence across the Pantanal between the overall habitat suitability for jaguars and the high-productivity areas for the livestock sector.

### Jaguar distribution model and wildfires

Our results demonstrate that, over the last four years, the incidence of wildfires has become gradually more severe in high-quality jaguar habitat (Fig. 1B; see Supplementary material). For example, the most recently mapped wildfires of 2020 comprised a significant portion of all critical jaguar habitat (r = 0.23, *p* < 0.001), whereas this spatial correlation was neutral in previous years (range =  − 0.07–0.03 in 2017–2019). With respect to the distribution of ecotourism lodges, only the 2020 wildfires showed a positive spatial correlation (r = 0.14, *p* < 0.001). Finally, for three of the last four years (2017, 2018, 2019) areas containing high cattle density were negatively correlated with the incidence of wildfires across the Pantanal.

## Discussion

Our results indicate that over 60% of the Pantanal ecosystem is considered highly suitable for the occurrence of healthy jaguar populations. This is even following an historical background of over 200 years of extensive cattle ranching, an activity that typically brings retaliation from humans, whereby felids are often killed in response to perceived risk of—or actual—livestock depredation^26,27^. Given the high percentage of suitable habitat, this indicates that traditional forms of low-yield economic use of the Pantanal (e.g., sustainable cattle ranching) is broadly compatible with the persistence of viable jaguar populations. This is intimately related to severe environmental limitations for the commercially viable livestock sector, imposed by the somewhat predictable annual cycle of droughts and floodwaters^28^. Vast lowland areas of the Pantanal remain inundated and isolated for many months each year, and therefore cannot be occupied by both cattle and humans. This land-use tradeoff, imposed by terrain elevation and hydrology, is central to juxtaposing wetland biodiversity conservation with financially workable agropastoral economics on higher ground.

On the other hand, ecotourism is not restricted by the limitations imposed by the Pantanal's highly seasonal hydrological regime. Ecotourism is widely considered as either an alternative or complementary economic activity for cattle ranching in the Pantanal^13,29^. The distribution of ecotourism lodges in the Pantanal has not changed very much over several decades. In fact, there is no obvious causality in the spatial distribution of lodges in relation to the present distribution of jaguars, as most of the lodges that now include jaguar tourism already operated commercially in terms of more general ecotourism prior to the onset of directed excursions targeting focal jaguar observations. Clearly, lodges sited in areas where jaguar habitat suitability is high are at an advantage as this is more likely to reward tourists for their investments and lead to positive publicity. The vast majority of these successful lodges are located where logistical infrastructure is available. This includes road access via the Transpantaneira Highway in the northern Pantanal and the BR-262 Highway in the south. Other lodges are located in areas where fishing tourism has been gradually replaced by wildlife tourism^14^. Extensive livestock ranching and ecotourism are compatible economic activities even within the same landholding^30^ and should be encouraged to facilitate the coexistence between jaguars and humans. Prospects of expanding ecotourism, however, would be very limited in remote regions^26^, which comprise approximately 30% of the Pantanal land area. We show that such areas often include high jaguar habitat suitability but currently lack any access infrastructure for lodges, thus indicating the limited potential expansion of jaguar-oriented tourism. Further development of ecotourism in the Pantanal requires government support, especially in the implementation and management of protected areas and support for sustainable infrastructure^14^. Another necessary precaution is to ensure that jaguar tourism does not become another threat to jaguars through poor management practices and over-interference on the species natural behaviour. Mismanaged ‘big cat’ tourism has already caused detrimental impacts on tigers (*Panthera tigris*) in India^31^ and lions (*Panthera leo*) in Africa^32^.

Our data clearly indicate highly favourable conditions for jaguar-focused, ecosystem-wide conservation in the Pantanal, given that the main economic activity in this biome (low-yield cattle ranching) shows very low spatial overlap with important jaguar strongholds. In addition to boosting local income, ecotourism increases diversified economic opportunities at the landholding and regional scales, and assists in the maintenance of both jaguar populations and their habitat. Perhaps the main role of ecotourism is to develop a “landscape of tolerance”, where jaguars can represent financial assets rather than costs, as shown in the northern Pantanal^17^. This landscape of tolerance, catalyzed by ecotourism, has already been proposed in other human-wildlife conflict scenarios involving large cats and livestock in Kenya^33^ and Botswana^34^. Savannah countries in Africa have been developing wildlife tourism for several decades, and can be used to assess the benefits that ecotourism can bring to large carnivore conservation^35,36^.

The current macroeconomic landscape of the Pantanal provides a favourable scenario for long-term jaguar conservation. However, it is important to consider that the Pantanal is a dynamic environment and its hydrological cycles presents an unpredictable supra-annual variation in flood intensity^37^. Recent studies considering climate change scenarios^38,39^ and deforestation in the Amazon^40^ predict a reduction in the annual rainfall that inundates the Pantanal during the wet season, which will further disrupt the hydrological cycle and lead to a seasonally drier Pantanal wetland. A major consequence of drying any vast wetland includes much higher ecosystem flammability^20^, as recently seen in the unprecedented wildfires of 2019 and 2020. We also show a growing multi-annual trend of wildfires affecting increasingly larger areas of the most suitable habitat for jaguar.

Beyond increasing the risk of wildfires, a drier Pantanal brings other threats for both jaguars and their prey base. The expansion of cattle ranching in the Pantanal is intrinsically connected with drier years^41^. The annual flood cycle imposes logistical limitations on cattle management, as it is not possible to build roads, fences, and corrals in areas that remain flooded for several months. Given the more frequent severe droughts in the Pantanal, such natural restrictions no longer prevent further expansion and/or intensification of cattle ranches. Larger numbers of cattle occupying the Pantanal region represent higher spatial overlap with areas most suitable for jaguars, which consequently increases the potential for human-jaguar conflict. Another indirect consequence of extreme droughts and wildfires that affects jaguar populations is the declining extent of permanently flooded areas suitable for semi-aquatic and aquatic prey, such as capybara (*Hydrochoerus hydrochaeris*) and caiman (*Caiman yacare*)^37^, which are closely associated with open-water environments. Some 43% of the regions recently affected by the 2020 wildfires occurred in previously flooded areas, with no records of fires in the previous two decades^22^.

Of all major Brazilian biomes, only the Amazon has a higher proportion of suitable jaguar habitat than the Pantanal^11^. The Amazon, however, is very remote and largely comprised of areas with relatively low economic use. In contrast, the most likely prospects for land-use economics in the Pantanal is currently sitting on a knife-edge upon which the status quo of nature conservation can either be maintained or spiral downwards, depending on the financial viability of the low-yield livestock sector. Projections of vegetation loss for the next 30 years indicate a scenario of an “arc of deforestation” progressively advancing into the Pantanal, while replacing traditional cattle ranching with high-yield croplands and ranching^19^.

Our results corroborate other studies that show the importance of the Pantanal for long-term jaguar conservation^10,16^. Traditional low-density cattle ranching has coexisted with jaguars for over 200 years and any discussion about the long-term viability of jaguar populations in the Pantanal inextricably involves supporting initiatives that can maintain the financial viability of traditional cattle ranching practices. Wildfires have also emerged as a new pervasive threat to natural ecosystems of the Pantanal that must be faced with fire-suppression measures, greater government enforcement preventing fire use during droughts, and rules of engagement regulating the timing of fire management of pastures^21,22,42^. Finally, the overall partition of land use across the Pantanal can support multiple economic benefits through jaguar-focused ecotourism. More specifically, ecotourism in the Pantanal can accrue direct conservation benefits for jaguars and other wildlife into the future, as it can help build not only a value-added landholding-scale economy, but embed social and cultural tolerance for jaguars before cattle ranching expands into newly suitable areas as the entire Pantanal biome becomes drier. Populations of the largest Neotropical felid can coexist side-by-side with cattle ranching, the oldest post-colonial economic activity in the Pantanal, thereby diversifying economic portfolios and encouraging the benign stewardship of private land managers.

## Methods

### Study area

**Figure 3:**
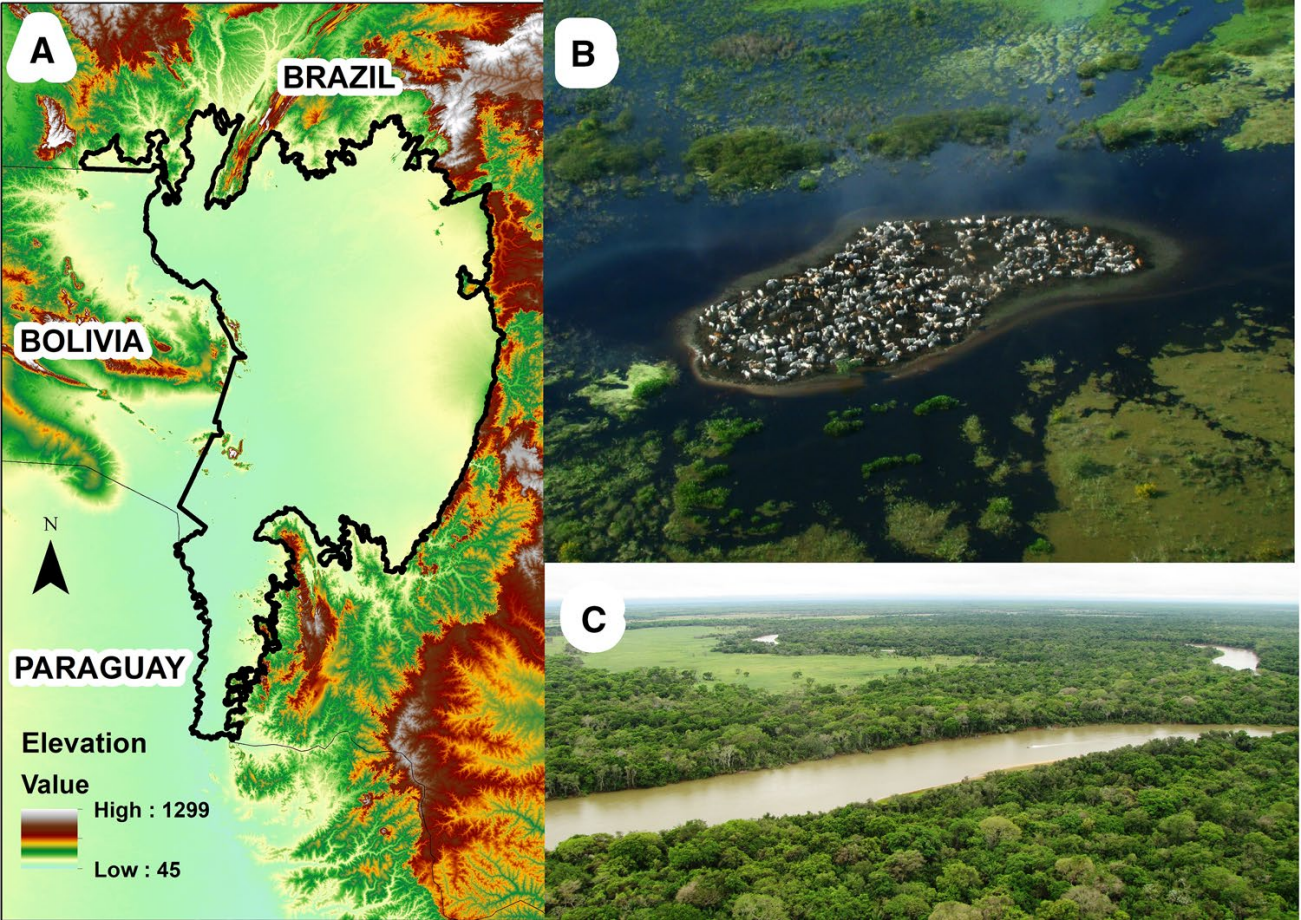
(**A**) Elevation map of the Pantanal Wetlands (Brazilian Pantanal, solid black line polygon), Map made in ArcGIS (v. 10.03; ESRI 2011) software; Aerial images of (**B**) a seasonally flooded grassland area with bovine cattle concentrated on higher elevation terrain (Photo: Rafael Hoogesteijn); and (**C**) a typical natural mosaic of forest and open grasslands of the Pantanal (Photo: Fernando Tortato).

The 179,300 km^2^ Pantanal biome, a geological depression of the Upper Paraguay River basin (Fig. 3A), is the largest inland tropical wetland on Earth. The Pantanal is widely celebrated for its highly visible wildlife value, and one of the few regions in South America where East African style wildlife safaris are possible. The Pantanal is located near the epicenter of South America, with most of its extent within Brazil and smaller portions located in Bolivia and Paraguay^13^. The Pantanal vegetation is a macromosaic influenced by the neighbouring central Brazilian wooded scrubland (Cerrado) savannah, the Amazon, and Chaco biomes (Fig. 3B,C). This wetland is shaped by the hydrological dynamics exerted by annual and supra-annual flood pulses^16^. For the purposes of this study, we considered only the Brazilian Pantanal, because it encompasses 78% of the entire ecoregion and our socioeconomic and biophysical databases did not include Bolivia and Paraguay.

### Jaguar distribution model (JDM)

This study used the jaguar presence database published in Brazil’s Jaguar Conservation Action Plan^11^ and 22 additional localities that we obtained from 2010–2017. We spatially rarified the presence database (SDM Toolbox^43^) at a distance of 6 km^44^ to preclude spatial autocorrelation, resulting in 147 unique presence points. We included the following biophysical layers as predictors in our models: bioclimate (gridded climate data; http://worldclim.org/version2^45^); topography (SRTM Digital Elevation Data; https://www2.jpl.nasa.gov/srtm/); and land cover (Global Land Cover Map; http://due.esrin.esa.int/page_globcover.php). We avoided multicollinearity by selecting only uncorrelated or weakly correlated variables (< 0.70) for modelling: elevation; land cover; annual mean temperature (*bio1*); mean diurnal temperature range (*bio2*); maximum temperature of the warmest month (*bio5*); mean temperature of the wettest quarter (*bio8*); precipitation of the driest month (*bio14*); and precipitation of the warmest quarter (*bio18*). All variables were resampled at a spatial resolution of 1 km^2^.

The JDM was developed using Maxent (v.3.4.1^46–48^), the most widely used SDM algorithm. Maxent estimates a target probability distribution by finding the probability distribution of maximum entropy, subject to a set of constraints that represent incomplete information about target distributions^46^. We set the default parameters in Maxent (convergence threshold of 1.0 × 10^−5^ = 0.000010, with 500 interactions and 10,000 background points, auto features), plus a variable importance analysis based on the jackknife, response curves, and random seed. The JDM was generated via bootstrapping methods with 10 random partitions and replacement, with 70% of the dataset used for training and 30% for testing the models. The result was a probabilistic model with pixel values ranging from 0.0 to 1.0. Higher suitability values represent higher probabilities of finding the species in the field. The output threshold of the JDM (i.e., habitat suitability) was interpreted to represent the probability of encountering one or more jaguars at a given site.

### Cattle density

Bovine cattle density (CD) was estimated using data available^13^, which considered both exotic pasture and natural grassland vegetation maps, the total pasture area per property, and the occupation rate of heads of cattle per hectare (i.e., stocking density) at each of the 22 municipal counties across the Pantanal. For our purposes, 10,000 points were randomly plotted throughout the Brazilian Pantanal while retaining a minimum distance of 3266 m between neighbouring points. This distance was based on the average size of rural landholdings in the Pantanal^13^. We then intersected the points with the CD map to extract density values for each of the 3631 rural properties. We derived a kernel map using a 25 km-radius and 900-m pixels, weighted in relation to the CD. Finally, we normalized the entire raster data within a range between 0.0 (minimum) and 1.0 (maximum) for each pixel.

### Ecotourism

We identified and mapped all non-urban lodges and hotels throughout the Pantanal using the compulsory federal registry of the Brazilian Ministry of Tourism (https://cadastur.turismo.gov.br/hotsite/). We conducted internet searches to identify lodges that were not yet registered in the federal system and confirm that all selected lodges indeed operated as ecotourism enterprises. Following identification, information on geographic location and private landholding boundaries were collected directly from the lodges via site visits and telephone calls. A kernel map was then generated based on these coordinates, with a radius of 25 km and pixels of 900 m. After this step, we normalized the raster data within a range between 0.0 (minimum) and 1.0 (maximum) for each pixel.

### Wildfires

Wildfire data were derived from a model that identified and assigned dates to all burnt areas. The burnt areas were identified from Chrono sequences of daily multispectral images retrieved from satellite imagery without the preprocessing need of cloud masking and image selection. The model used input data from the 750 m bands of VIIRS that was resampled to a 0.01° spatial resolution grid. The derived burned areas were validated against higher resolution reference maps and compared to the global burned area datasets MCD64A1 Collection 6 and FireCCI51^49^. These spatial data were made available by the Environmental Satellite Applications Laboratory of the Federal University of Rio de Janeiro^50^. For our study, we used shapefiles depicting all annual burnt areas of the entire Brazilian Pantanal for four consecutive years (2017, 2018, 2019 and 2020).

### Data analysis

After developing each layer (i.e., jaguar habitat suitability; cattle stocking density; density of ecotourism lodges; and 2017–2020 burnt areas), we divided the Brazilian Pantanal into 4951 hexagonal cells, each of which corresponded to the average size of an operational cattle ranch in the Pantanal^13^. For each hex-cell, we extracted the average pixel value for each raster (jaguar habitat suitability; cattle density; and density of ecotourism lodges). For the wildfire data, we extracted the proportional area that was burnt in each hex-cell in each of the four years (2017–2020). To facilitate the analysis, all data were log-transformed. A Pearson correlation matrix was then performed to examine the relationships between the seven spatial layers at the scale of hex-cells. Permutation tests were used to further explore pairwise spatial differences at the hex-cell scale (*N* = 4951) between JDM, cattle suitability, and ecotourism potential.

Our null model used randomly shuffled observed values without replacement while keeping sample sizes constant for 5000 iterations. We compiled difference values for each iteration to create 5000 distributions of potential differences. To obtain a probability value, these differences were compared to their respective probability distributions from the permutations. Spatial layers were processed using QGIS (v. 3.16.5; QGIS Development Team 2021) and ArcGIS (v. 10.03; ESRI 2011) software. Data were analyzed using the “tidyverse” workflow^51^ and the “infer”^52^ package in R (R Core Team 2021). To formally assess spatial autocorrelation, we used the “SpatialPack” R package^53^, which quantifies the spatial association between any two defined processes on a finite subset of a spatial plane.

## Supplementary Information

Supplementary Information.
