# Supplementary material for: Reconciling biome-wide conservation of an apex carnivore with land-use economics in the increasingly threatened Pantanal wetlands
Source: Sci Rep. 2021 Nov 23;11:22808. doi: 10.1038/s41598-021-02142-0 (PMC8611020; doi:10.1038/s41598-021-02142-0)
Supplement: Supplementary file 1 — Supplementary Information. [file 41598_2021_2142_MOESM1_ESM.docx]

Supplementary information for

**Reconciling biome-wide conservation of an apex carnivore with land-use economics in the increasingly threatened Pantanal wetlands**

Fernando R. Tortato^1^*, Rafael Hoogesteijn^1^, Allison L. Devlin^1, 6^, Howard B. Quigley^1^, Fábio Bolzan^2^, Thiago J. Izzo^3^, Katia M. P. M. B. Ferraz^4^, Carlos A. Peres^5,7^

1. Panthera, 8 West 40th Street, 18th Floor, New York, USA

2. Departamento de Ecologia, Instituto de Biologia, Universidade Federal do Mato Grosso do Sul, Campo Grande, MS, Brazil

3. Departamento de Ecologia e Botânica, Universidade Federal de Mato Grosso, Cuiabá, Mato Grosso, Brazil

4. University of São Paulo, Forest Science Department, Luiz de Queiroz College of Agriculture, Piracicaba, SP, Brazil

5. Centre for Ecology, Evolution and Conservation, School of Environmental Sciences, University of East Anglia, Norfolk, Norwich, UK

6. Wildlife Biology Program, W.A. Franke College of Forestry & Conservation, University of Montana, Missoula, MT 59812 USA

7. Instituto Juruá, Rua das Papoulas 97, Manaus, Brazil

Corresponding Author: ftortato@panthera.org


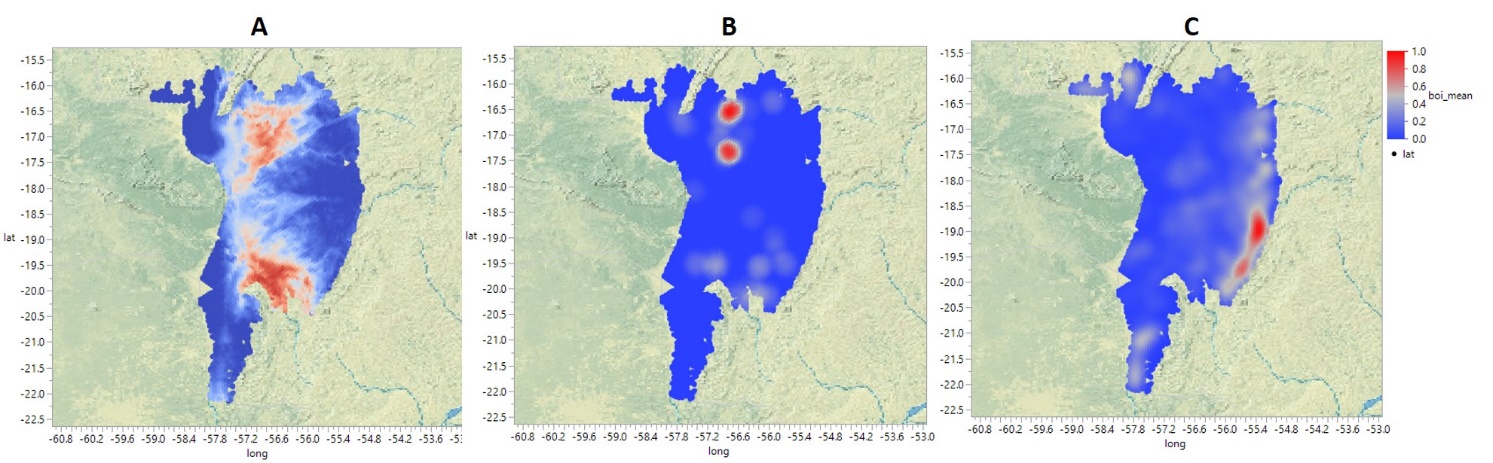


Supplementary figure 1. Maps of the Brazilian Pantanal where: A represents the habitat suitability model for jaguar (*Panthera onca*); B represents ecotourism lodge density model; and C represents livestock density model. The maps were developed by SAS software (SAS Institute Inc., Cary, NC, USA) (https://www.sas.com/).


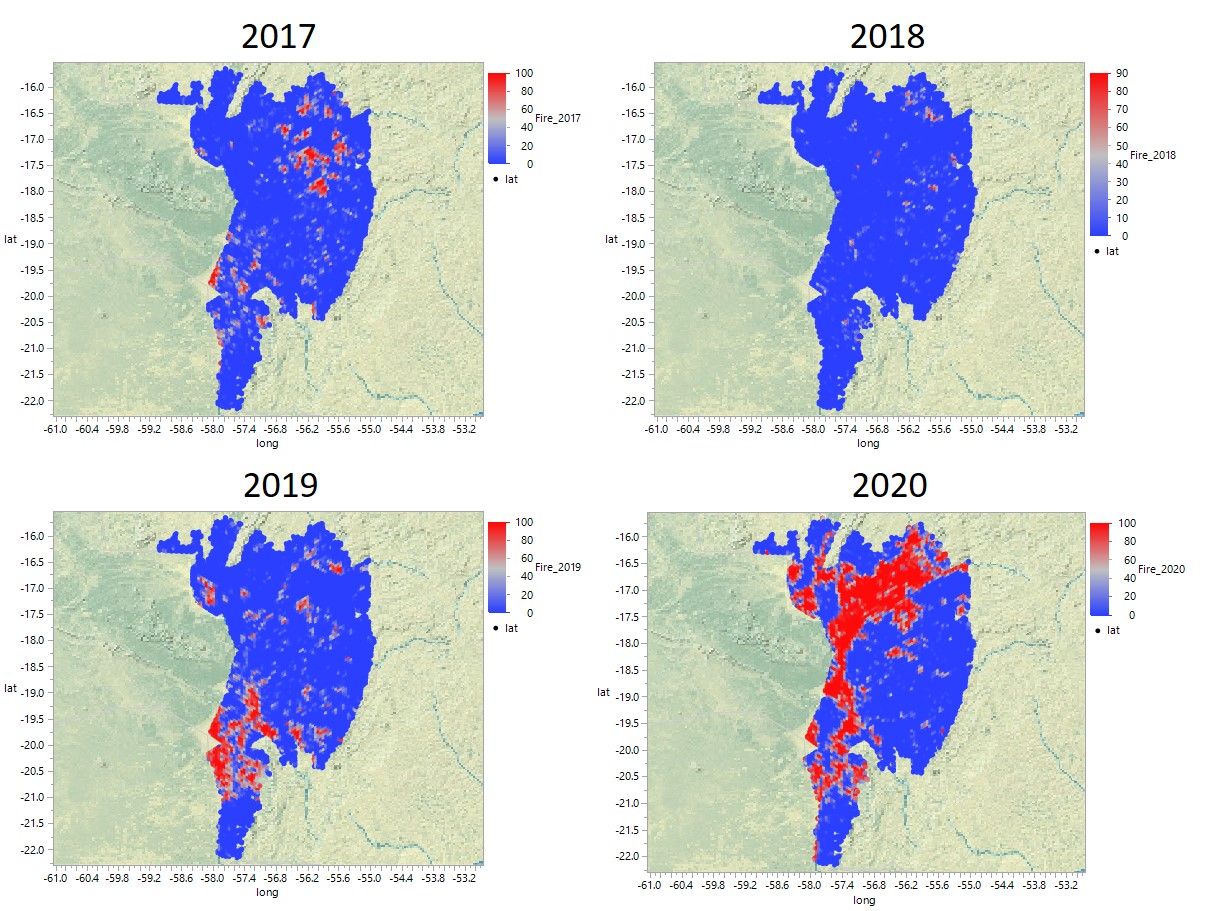


Supplementary figure 2. Spatial distribution of wildfires in the Pantanal for the years 2017, 2018, 2019 and 2020 with red colours indicating areas of fire incidence. The maps were developed by SAS software (SAS Institute Inc., Cary, NC, USA) (https://www.sas.com/).
